# Gaze Compensation as a Technique for Improving Hand–Eye Coordination in Prosthetic Vision

**DOI:** 10.1167/tvst.7.1.2

**Published:** 2018-01-05

**Authors:** Samuel A. Titchener, Mohit N. Shivdasani, James B. Fallon, Matthew A. Petoe

**Affiliations:** 1 The Bionics Institute of Australia, East Melbourne, Australia; 2 Department of Medical Bionics, University of Melbourne, Parkville, Australia

**Keywords:** visual prosthesis, eye tracking, retinal implant, spatial localization, gaze compensation

## Abstract

**Purpose:**

Shifting the region-of-interest within the input image to compensate for gaze shifts (“gaze compensation”) may improve hand–eye coordination in visual prostheses that incorporate an external camera. The present study investigated the effects of eye movement on hand-eye coordination under simulated prosthetic vision (SPV), and measured the coordination benefits of gaze compensation.

**Methods:**

Seven healthy-sighted subjects performed a target localization-pointing task under SPV. Three conditions were tested, modeling: retinally stabilized phosphenes (uncompensated); gaze compensation; and no phosphene movement (center-fixed). The error in pointing was quantified for each condition.

**Results:**

Gaze compensation yielded a significantly smaller pointing error than the uncompensated condition for six of seven subjects, and a similar or smaller pointing error than the center-fixed condition for all subjects (two-way ANOVA, *P* < 0.05). Pointing error eccentricity and gaze eccentricity were moderately correlated in the uncompensated condition (azimuth: *R*^2^ = 0.47; elevation: *R*^2^ = 0.51) but not in the gaze-compensated condition (azimuth: *R*^2^ = 0.01; elevation: *R*^2^ = 0.00). Increased variability in gaze at the time of pointing was correlated with greater reduction in pointing error in the center-fixed condition compared with the uncompensated condition (*R*^2^ = 0.64).

**Conclusions:**

Eccentric eye position impedes hand–eye coordination in SPV. While limiting eye eccentricity in uncompensated viewing can reduce errors, gaze compensation is effective in improving coordination for subjects unable to maintain fixation.

**Translational Relevance:**

The results highlight the present necessity for suppressing eye movement and support the use of gaze compensation to improve hand–eye coordination and localization performance in prosthetic vision.

## Introduction

Visual prostheses aim to provide artificial vision to blind patients by using implanted electrodes to electrically stimulate the retina,^1–4^ optic nerve,^5^ thalamus,^6^ or visual cortex,^7^ evoking localized visual percepts. The location of the percept within the patient's egocentric spatial map is known to move in parity with the orientation of the eyes.^1,7,8^ This apparent movement is because eye position plays an important role in the integration of retinotopic visual signals into a consistent spatial map,^9^ even after blindness.^10^

For tasks of coordination it is important that the percept location properly reflects the real world. This requires the orientation of the image sensor to be directly coupled with eye position; however, most present devices use an external camera of fixed orientation, divorcing the camera axis from the pupillary axis.^1–3^ Recipients of these devices must rely exclusively on head movements to direct their field of view. While retinal implants have been shown to assist in hand–eye coordination tasks^2,11–13^ it is likely that the decoupling of the camera and pupillary axes negatively affects performance on the tasks.

Currently, patients are trained to suppress eye movements at all times in order to maintain alignment between the camera and pupillary axes^14,15^ but the accessibility of this technique is questionable. Patients have little intuition of the orientation of their eyes^8^ and have difficulty suppressing eye movements, particularly those associated with nystagmus. We have previously found that suprachoroidal implant recipients made significant eye movements in response to stimuli during a static image localization task, despite being instructed not to.^16^ A separate study in Argus II recipients found that camera-gaze misalignments occurred frequently during a visual search task, often due to the vestibulo-ocular reflexive movements that occur naturally during head scanning, and that patients rely on a series of complex head movements to properly localize objects in daily life.^8^ Some have suggested that percept localization is so difficult that many patients simply use their devices as light detectors, ignoring any retinotopic information and instead relying solely on head and neck orientation.^1,11,17^

Other visual prostheses forego the external camera and instead use implanted photodiode arrays, such as the Alpha IMS (Retina Implant AG, Reutlingen, Germany) subretinal implant.^4^ In these devices, electrode activity is modulated by the light that is naturally incident to the eye, enabling naturalistic eye scanning. Studies with Alpha IMS recipients have shown that patients exhibit “qualitatively normal” oculomotor behavior when the hardware permits eye scanning.^18^ Restoring naturalistic eye scanning in camera-based retinal and cortical implants is desirable as it has implications for perceptual localization and hand–eye coordination, and would reduce the cognitive burden on the recipient by facilitating more intuitive interaction with the technology. Implantable intraocular cameras have been proposed as one way of achieving this,^19–21^ but to our knowledge the clinical feasibility of this approach has not been established. Others have proposed tracking the eye position and dynamically shifting the region-of-interest (ROI) inside a wide field of view image to compensate for eye movements as they occur.^22,23^ This technique, which we term “gaze compensation,” is the more immediately applicable and clinically relevant technique.

Existing studies have examined the benefits of gaze compensation in prosthetic vision. In a previous study on suprachoroidal retinal implant recipients, we showed that gaze compensation improved performance in a static image localization task but we did not assess hand–eye coordination specifically.^24^ Similarly, McIntosh^23^ showed that subjects under simulated prosthetic vision performed better in a reach-and-grasp task and a visual search task when foveation was restored; however, significant results were found only at high phosphene densities, possibly because the tasks were too difficult to perform at low resolution even with gaze compensation.

Other studies have reported eye position as a confounding factor in hand–eye coordination. Sabbah et al.^8^ tested the accuracy of epiretinal Argus II patients in a target localization task when the eyes were purposefully held in an eccentric position. They reported that pointing was skewed toward the direction of eye displacement; however, the analysis was limited to directionality and did not quantify the effect of eye displacement magnitude. In a separate study, Argus II patients indicated the location of percepts generated by direct-to-array stimulation during forced eccentric eye movements. After estimating the effect of eye movement, the authors inferred the retinotopic placement of electrodes from the pointed location.^25^ A simulated prosthetic vision study in a visually impaired subject found that nystagmus adversely affected performance on a hand–eye coordination task when phosphenes moved in parity with the eyes.^22^ Two preliminary reports regarding experiments in Argus II recipients (Caspi et al. *IOVS* 2017;58:ARVO E-Abstract 4192) and simulated prosthetic vision (Hozumi et al. *IOVS* 2016;57:ARVO E-Abstract 1958), respectively, have demonstrated reduced pointing error in a target localization task when gaze compensation was used. Finally, changes in the optimal camera alignment in Argus II patients have been shown to correlate with long-term changes in eye orientation (Barry et al. *IOVS* 2017;58:ARVO E-Abstract 4687). It is clear that a relationship between eye position and hand–eye coordination exists, but to our knowledge the specific effect of gaze eccentricity on coordination has not been characterized in any of the existing literature.

The present study aimed to test the effectiveness of gaze compensation for improving hand–eye coordination in visual prosthetic recipients. We used a prosthetic vision simulator, based on the classic scoreboard model of phosphene vision, with built-in eye tracking to simulate prosthetic vision with and without gaze compensation. Further, we aimed to characterize the relationship between eye position and pointing error in a target localization task under simulated prosthetic vision in order to better understand the effect of eye movements on hand–eye coordination. In contrast to the studies by Caspi et al.^25^ and Sabbah et al.,^8^ any pupil eccentricity was spontaneously occurring rather than experimenter controlled. We hypothesized that pointing error in the gaze-compensated condition would be significantly smaller than in the uncompensated condition and comparable to an idealized condition in which phosphenes never moved and camera-gaze misalignments did not arise from eye movement. We further hypothesized that the magnitude and directionality of pointing error would be correlated to eye position, but that these correlations would diminish with gaze compensation.

## Methods

### Subject Selection

Seven volunteers aged 22 to 31 participated in the experiment. All subjects had normal or corrected-to-normal vision and had no relevant medical history, and informed consent was obtained for all subjects. The research adhered to the tenets of the Declaration of Helsinki and was approved by The University of Melbourne School of Health Sciences Human Ethics Advisory Group (HREC 1647240).

### Phosphene Rendering for Simulated Prosthetic Vision

**Figure 1:**
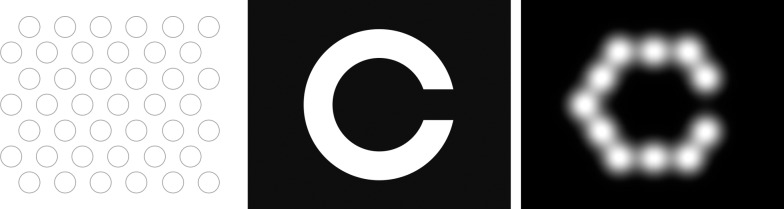
Phosphene rendering for simulated prosthetic vision using the abstract model described by McCarthy et al.^29^ Left: electrode layout. Center: input image. Right: phosphene rendering produced by sampling the input image at the electrode locations.

Real-time phosphene rendering for simulated prosthetic vision was achieved using an abstract model of phosphene vision previously described by McCarthy et al.^26^ Briefly, phosphenes appeared in a predefined layout as white spots whose intensity was greatest in the center and decayed with radial distance according to a Gaussian profile with standard deviation proportional to the peak intensity. Phosphene intensities were calculated according to the minimal vision processing scheme described by Barnes et al.,^27^ whereby each phosphene intensity was calculated using a projection of 42 electrodes onto the input image and the peak intensity of each phosphene was set to a quantized value (8 levels) of the underlying pixel in the input image. The phosphene layout was modeled after the electrode layout of Bionic Vision Australia's second-generation 44-channel retinal implant,^28^ with 42 phosphenes arranged in a hexagonal grid (Fig. 1).

### Simulated Prosthetic Vision Apparatus

**Figure 2:**
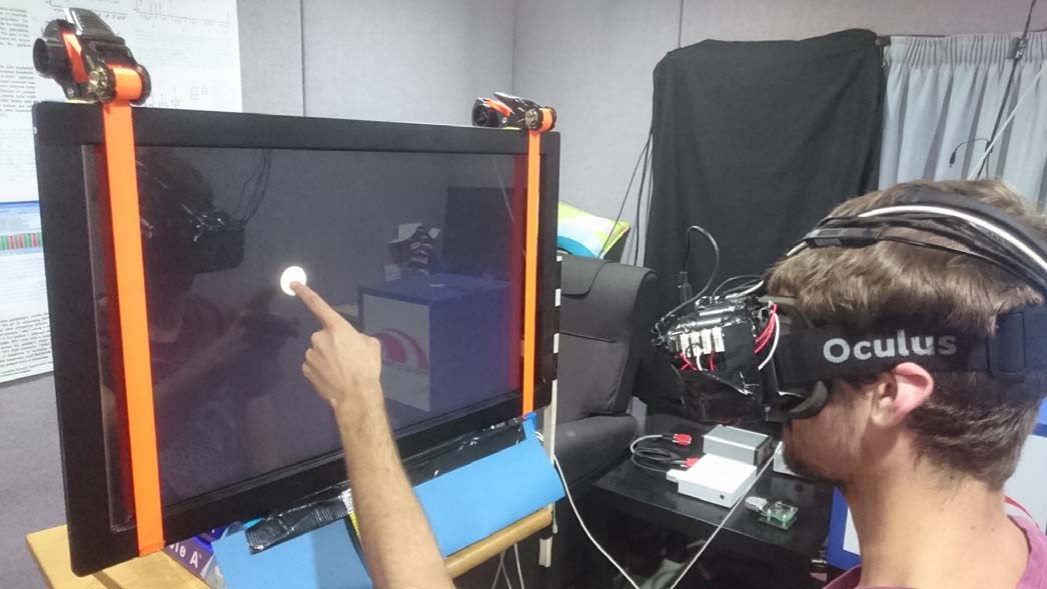
Experiment set-up. Targets were displayed on a 30-inch touchscreen at a viewing distance of 40 cm. Subjects wore a simulated prosthetic vision headset that included a front-facing camera, head motion tracker, and eye tracker.

Subjects wore a virtual reality headset (Rift DK2; Oculus Inc., Irvine, CA, USA) with a wide field of view (FOV) camera (Logitech c390e, FOV: 80.7° × 50°, 30 fps; Logitech, Lausanne, Switzerland) mounted to the headset in front of the left eye (Fig. 2). Images from the camera were prewarped to eliminate lens distortion. A 15° × 15° ROI (based on a conservative retinal projection of the electrode array^29^) was sampled from each frame to be rendered as a phosphene image and presented on the headset display to the left eye only. The subject could redirect the camera axis by moving their head. Phosphene rendering latency was 50 ms and the display refresh rate was 90 Hz, yielding latencies between 50 and 61 ms for the display to reflect a change in camera image.

### Tracking Eye and Head Position

**Figure 3:**
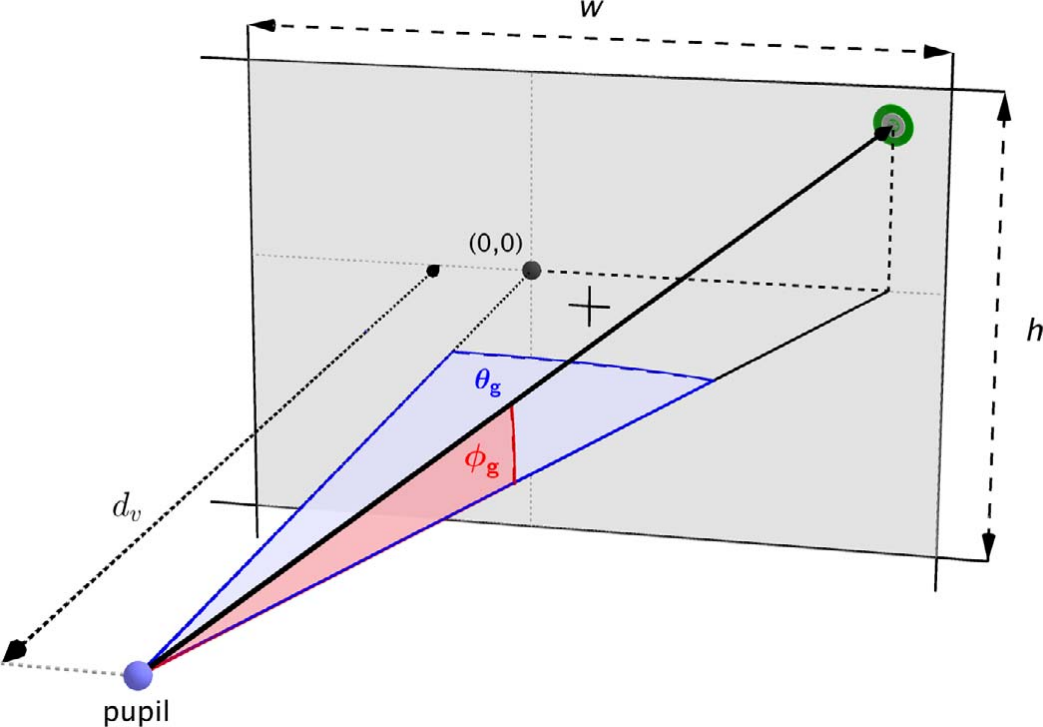
Eye tracker calibration. The subject was required to fixate on a series of calibration targets (green) on the headset display. The heavy black line shows the direction of gaze. The eye tracker software produced a calibration that mapped the pupil location to headset display pixel coordinates. Then, by using the known screen geometry (display width, w; display height, h; viewing distance, d_v_) and estimated gaze origin (0, 0), gaze locations in pixel coordinates were transformed to azimuth (θ_g_) and elevation (φ_g_) angles in a head head-centered coordinate system relative to the natural gaze origin. The black plus indicates the center of the display, which was not necessarily aligned with the natural gaze origin. Real-world locations were expressed in the same head-centered coordinate system by accounting for the instantaneous measured head position.

An infrared eye tracker (Arrington Research, Scottsdale, AZ, USA) mounted inside the headset recorded the position of the left pupil at 60 Hz, to an accuracy of ±0.5°. Blinks and poor quality data points were identified and discarded based on a pupil size and shape criterion reported by the eye tracker software. A 16-point fixation target calibration procedure mapped pupil locations within the eye tracker camera image to pixel locations on the headset display. The location of the natural gaze origin relative to the headset display was estimated post hoc for each subject from the distributions of measured eye azimuths and elevations during the stimulus-absent periods between experiment trials. Gaussian curves were fitted to the two distributions, and the gaze angles at the peak of each curve were taken as the natural gaze origin. Then, using the known geometry of the headset, gaze locations in pixel coordinates were transformed to degrees of visual arc relative to the natural gaze origin in a head-centered coordinate system. The combined eye azimuth (θ) and elevation (φ) are referred to as the “gaze angle.” Figure 3 illustrates the calibration process.

A motion tracker (trackSTAR; Ascension Technology Corp., Shelburne, VT, USA) on the headset recorded head position and bearing at 20 Hz. By accounting for the instantaneously measured head position the locations of real-world objects were also expressed in the head-centered coordinate system, allowing for direct comparison between eye position and world locations.

### Phosphene Movement Conditions

The simulator could optionally use eye position measurements in a closed loop configuration to retinally stabilize the phosphenes. Additionally, the input image sampling ROI could be shifted in parity with the measured gaze angle (gaze compensation). Three conditions were tested:

1. Uncompensated: modeled a retinal implant without gaze compensation. Phosphenes were retinally stabilized and the camera image ROI remained fixed in the center of the image. Head scanning was the only means of directing the FOV, and eye movements could introduce camera-gaze misalignments.
2. Gaze-compensated: modeled a retinal implant with gaze compensation. Phosphenes were retinally stabilized and gaze compensation was applied to continuously transpose the input image sampling ROI. The camera image was mapped to the headset display such that the center of the image corresponded to the center of the display. Head scanning and eye scanning could both be used to direct the FOV.
3. Center-fixed: a control condition is which the phosphenes never moved. The phosphene array and input image sampling ROI remained fixed in the centers of the headset display and the camera image respectively, regardless of any eye movement. Subjects were able to foveate on any part of the FOV; however, eye movements did not cause the phosphene array shift relative to the camera image. Head scanning was the only method of directing the FOV.

The latency for the phosphene display to respond to an eye movement ranged from 52 to 80 ms (60-Hz eye tracker acquisition + 2-ms eye tracker processing latency + 50-ms phosphene rendering + 90-Hz display refresh), which may have been perceptible as a slight lag in response.

### Target Localization Task

Subjects wore the prosthetic vision simulator and sat 40 cm in front of a touchscreen monitor in a darkened room. The monitor (Dell U3011; Dell, Round Rock, TX, USA) measured 30-inches diagonally with a 16:10 aspect ratio, equating to a FOV of 78° × 54°. Subjects were free to move their heads but were encouraged not to move their upper bodies so as to maintain a constant distance to the monitor. In each trial of the task, a single stationary white circular target with a diameter of 5° appeared in a random location on the monitor, restricted to the central 52° × 34° region, against a black background. The appearance of the target was accompanied by an audio tone that signaled the start of the trial. Subjects were instructed to search for the target and touch it with a finger of their dominant hand to the best of their ability with no restriction on time, and were permitted to rest their nondominant hand on the table to orient themselves throughout the task. A different audio tone signaled when a touch had been successfully registered by the touchscreen monitor, ending the trial. Figure 2 shows the experiment set up.

One of the three simulator conditions (uncompensated, gaze-compensated, or center-fixed) was selected at random for each trial. Subjects were not briefed on the parameters of the different conditions. Trials were executed in blocks of 10, and before each block an eye tracker slip-correction sequence, in which the subject briefly fixated a single calibration target, was executed to account for any relative movement between the eye tracker camera and the subject's eye. The touch location, target location, response time, and the eye and head positions during the trial were recorded. Subjects could stop or request a break at any time and breaks were enforced after each 100 trials. Prior to beginning the experiment, subjects performed three to 10 trials using normal vision to familiarize themselves with the task. The primary stopping condition was 240 trials in total and at least 50 trials in each condition. In a debriefing session following the experiment, subjects were asked to describe any strategies they had used in the task.

### Data Analysis

The primary measure of performance on the task was the pointing error, which characterized hand–eye coordination. Pointing error was measured in degrees of visual arc between the center of the circular target and the location touched by the subject. This enabled direct comparisons between the gaze angle, target, and touch in a common head-centered coordinate system relative to the natural gaze origin (Fig. 3). The gaze angle at the time that the subject touched the screen is referred to as the “response gaze.”

Task performance, measured in terms of pointing error and response time, was compared between simulator conditions using two-way ANOVAs with subject and condition as factors. We then examined the relationship between response gaze and pointing error in terms of magnitude and directionality to determine whether eccentric gaze was correlated with poor performance in the gaze-compensated and uncompensated conditions. Finally, the distribution of eye positions recorded during the experiment was examined to quantify the typical range of eye movements during the localization task.

The center-fixed condition was used as a control for any systematic biases in pointing error, because phosphenes were stationary and eye position was not expected to affect pointing error in this condition. Two sources of bias were considered:

1. Gaze offset bias: any offset between the natural gaze origin and the center of the headset display, which was mapped to the center of the camera image, introduced a constant displacement between the percept (as seen by the subject) and the target. The gaze offset bias existed only in the center-fixed and gaze-compensated conditions, because in the uncompensated condition the center of the camera image was mapped to the fovea rather than to any particular area of the headset display.
2. Open-loop pointing bias: arises from the lack of visual feedback with which to guide the pointing motion,^30^ as well as from the spatial separation between the image sensor and the headset display. This bias is likely to be present equally in all three conditions as it is intrinsic to the individual subject and to the physical dimensions of the simulator apparatus.

Assuming a 1:1 correlation between gaze offset and pointing error, the gaze-offset bias was equal to the visual arc between the natural gaze origin and the display center. This value was subtracted from the recorded touch location for each trial in the center-fixed and gaze-compensated conditions only. The remaining systematic bias in the center-fixed condition is attributed to open-loop pointing bias and was subtracted from the recorded touch locations in all conditions. All pointing errors presented have been treated in this way unless otherwise stated.

## Results

**Figure 4:**
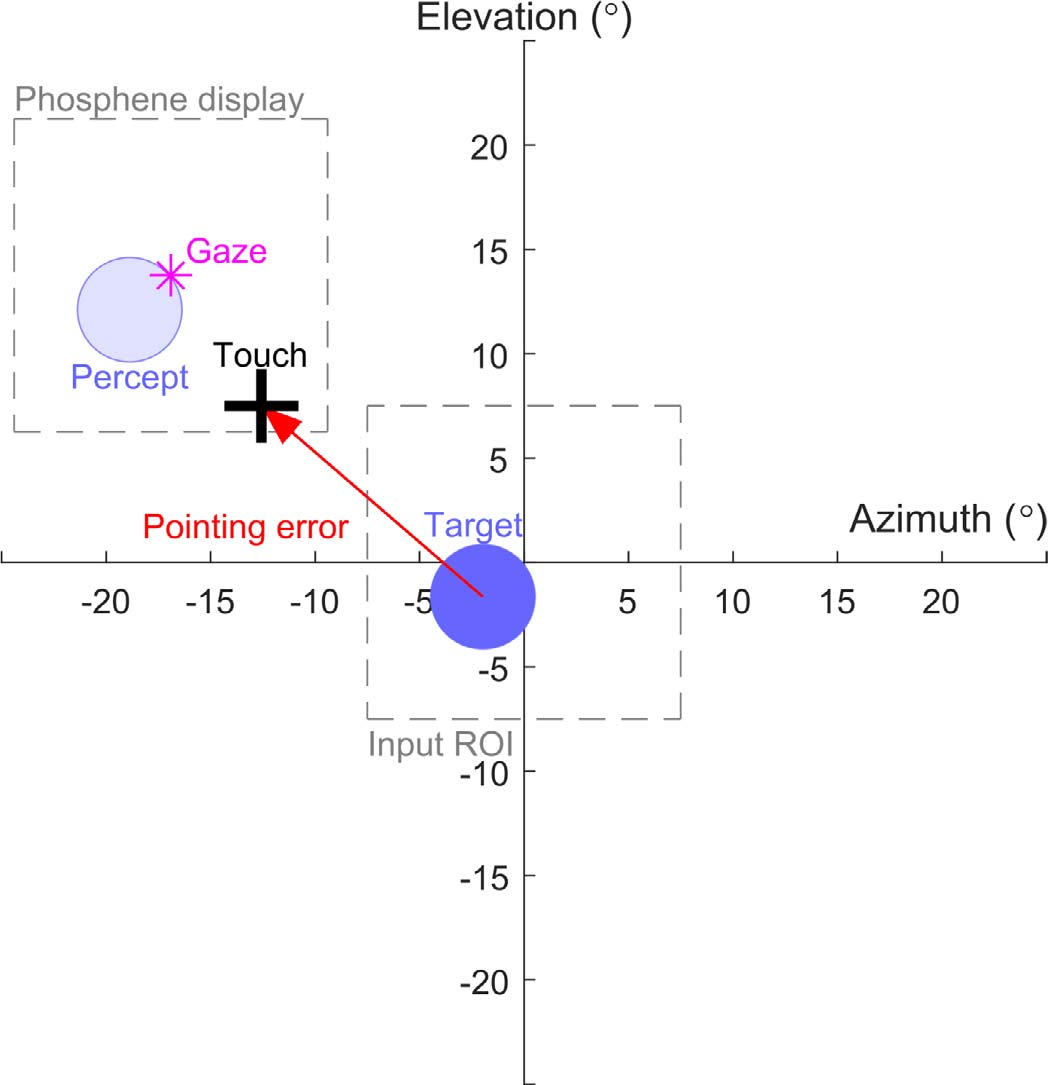
Results from a single trial for subject S6 in the uncompensated condition. The input image region-of-interest is sampled from a central location while the phosphene display appears at the gaze location. The location of the 5° target (dark blue circle), the location touched by the subject (black plus symbol), the gaze location (magenta star), and the location of the percept representing the target (transparent blue circle) are all expressed in degrees of visual arc in the head-centered coordinate system. The pointing error (red arrow) is measured between the target location and the touch location. Note the percept of the target has moved in parity with the subject's gaze resulting in a pointing error in the direction of the percept despite the camera being pointed toward target.

All subjects were able to complete the task, and each subject performed between 57 and 89 trials under each condition. Figure 4 shows an example result from a single trial for subject S6 in the uncompensated condition.

### Systematic Bias in Pointing Error

**Figure 5:**
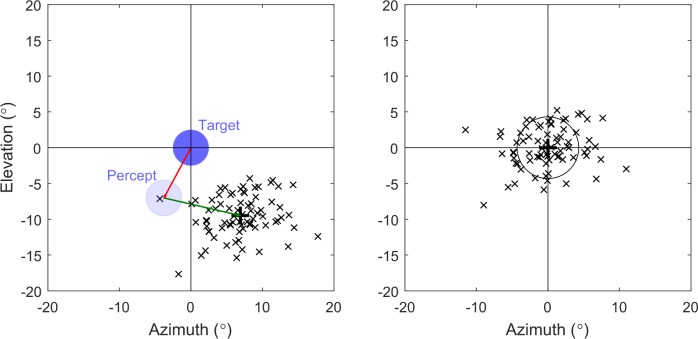
Systematic bias in pointing error for subject S3 in the center-fixed condition. Left: touch locations relative to the target location (black crosses, with centroid indicated by the black plus symbol) are biased downward and rightward of the target. The bias is composed of gaze-offset bias (red arrow), caused by the misalignment between the fixed phosphene array and the gaze origin, and open-loop pointing bias (green arrow). Right: pointing error for the same subject with bias subtracted.

Figure 5 demonstrates systematic bias in pointing error for subject S3 in the center-fixed condition, showing the separate contributions of gaze offset bias and open-loop pointing bias. Among the cohort, gaze offset magnitudes ranged from 3.3° to 15.9°, open-loop pointing bias magnitudes ranged from 9.6° to 14.7°, and total bias magnitudes ranged from 4.9° to 17.6°.

### Performance Measures

**Figure 6:**
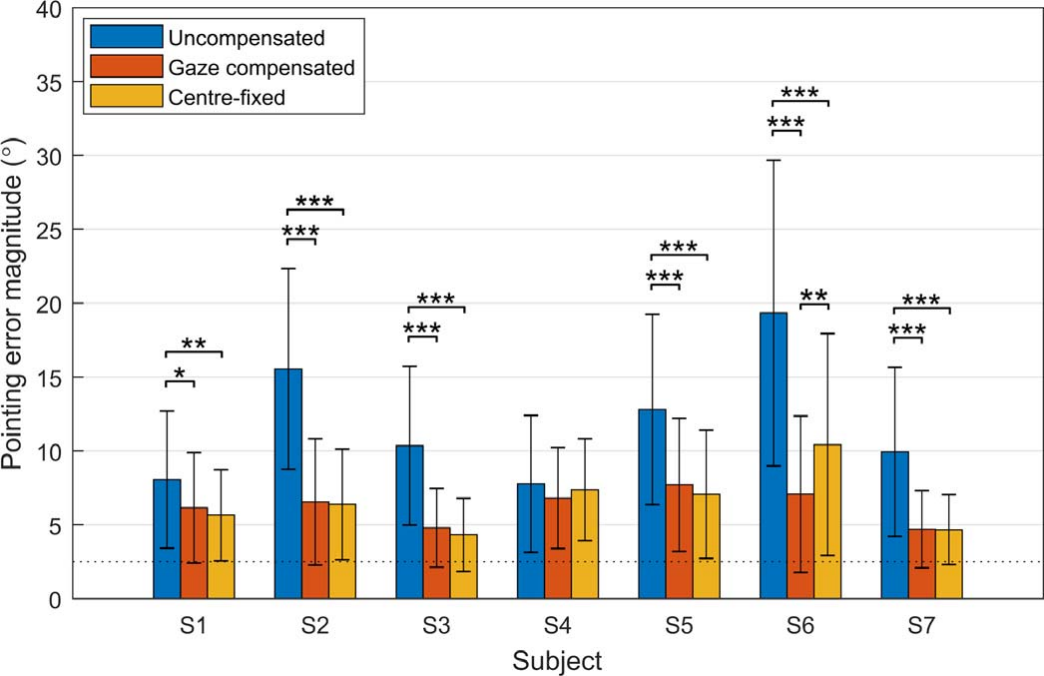
Mean pointing error magnitude in each condition for each subject. Error bars: standard deviations; asterisks denote a significant difference between two conditions (*P < 0.05; **P < 0.01; ***P < 0.001), and the dashed line indicates the radius of the target. Pointing error was significantly greater in the uncompensated condition than in the gaze-compensated and center-fixed conditions for six of seven subjects. A significant difference between the gaze-compensated and center-fixed conditions was observed only for S6.

The data for pointing error across conditions and subjects were heteroscedastic and had nonnormally distributed residuals. For analysis of pointing error, we performed a rank transform in conjunction with Welch's ANOVA, which does not assume homoscedasticity and is insensitive to nonnormality for large sample sizes.^31^ A separate Welch's ANOVA was performed for each subject using a Bonferroni-adjusted alpha level (α = 0.05/7, or 0.007) and the Games-Howell procedure for multiple comparisons. For six of seven subjects, pointing error magnitude (Fig. 6) was significantly greater in the uncompensated condition than the gaze-compensated and center-fixed conditions (S1 *P* < 0.05; S2, S3, S5, S6, S7 *P* < 0.001). No significant difference was detected between the gaze-compensated and center-fixed conditions for any subject except S6, for whom the center-fixed condition resulted in a larger pointing error (*P* < 0.003).

The mean pointing error across all subjects after correcting for systematic bias was 6.6° ± 0.2° in the gaze-compensated condition, 6.7° ± 0.2° in the center-fixed condition, comparable to 5.0° ± 1.6° for simulated ultra-low vision subjects reported by Endo et al.^33^ Mean pointing error in the uncompensated condition across all subjects was 12.1° ± 0.3°. A previous study in simulated prosthetic vision reported higher mean pointing error for gaze-compensated and uncompensated viewing, possibly because they had not corrected for bias in pointing (Hozumi et al. *IOVS* 2016;57:ARVO E-Abstract 1958).

Response times varied from 0.85 to 109.05 seconds. There was a significant effect of condition on response time for subject S1 only (Separate Welch's ANOVAs on ranks for each subject with Bonferroni correction and Games-Howell's multiple comparisons procedure); S1 had significantly shorter response times in the center-fixed condition than in the gaze-compensated (*P* < 0.001) and uncompensated (*P* < 0.001) conditions. Welch's ANOVA was selected because the data were heteroscedastic and has nonnormally distributed.

### Eccentric Gaze as a Confounding Factor

**Figure 7:**
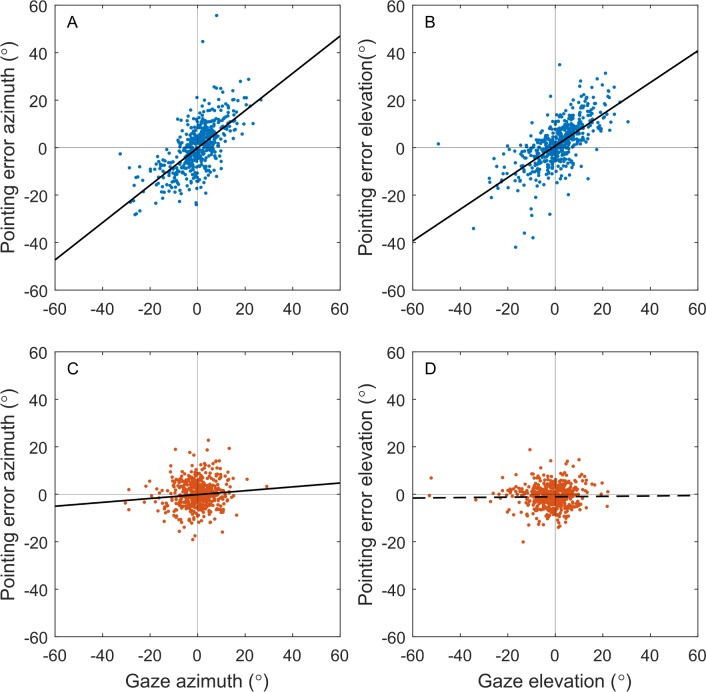
Pointing error against response gaze for the uncompensated condition (top row) and the gaze-compensated condition (bottom row). The azimuth dimension (left column) is analyzed separately from the elevation dimension (right column). Data are aggregated across all subjects. Solid black lines indicate significant linear trends (P < 0.05), and dashed black lines indicate insignificant trends.

Our second hypothesis stated that the larger pointing error in the uncompensated condition was specifically attributed to noncentral gaze. We investigated this by testing correlation between pointing error and response gaze for the gaze-compensated and uncompensated conditions using least-squares linear regression analysis (Fig. 7). Data from all subjects were analyzed collectively. The azimuth and elevation were analyzed separately to preserve both the magnitude and directionality of the pointing error vector. Although the residuals were found to be nonnormally distributed in all four cases, linear regression is robust against nonnormality, particularly with large sample sizes.

In the uncompensated condition, there was a moderate correlation between gaze and pointing error in both dimensions (Fig. 7A, azimuth: *R*^2^ = 0.47, slope = 0.79, *P* < 0.001; Fig. 7B, elevation: *R*^2^ = 0.51, slope = 0.67, *P* < 0.001), with the pointing error smallest when gaze was central and largest when gaze was eccentric. In the gaze-compensated condition, there was a statistically significant but extremely weak correlation between the response gaze azimuth and pointing error azimuth (Fig. 7C, azimuth: *R*^2^ = 0.01, slope = 0.08 *P* < 0.05), and no significant correlation in the elevation dimension (Fig. 7D, elevation: *R*^2^ = 0.00, slope = 0.01, *P* = 0.72).

As a secondary measure, the correlation between the vector angles of response gaze and pointing error was tested using circular correlation analysis as described by Jammalamadaka et al.^32^ and implemented in the CircStat toolbox for Matlab.^33^ This method purely examines directional information, discarding magnitude. All error-free trials (i.e., touch point within target boundary, 2.5° from target center) were excluded. A significant correlation between the directionalities of gaze and pointing error was observed in the uncompensated condition (Jammalamadaka's *r* = 0.58, *P* < 0.001) but not the gaze-compensated condition (*r* = −0.00, *P* = 0.93).

### Scanning Strategies

**Figure 8:**
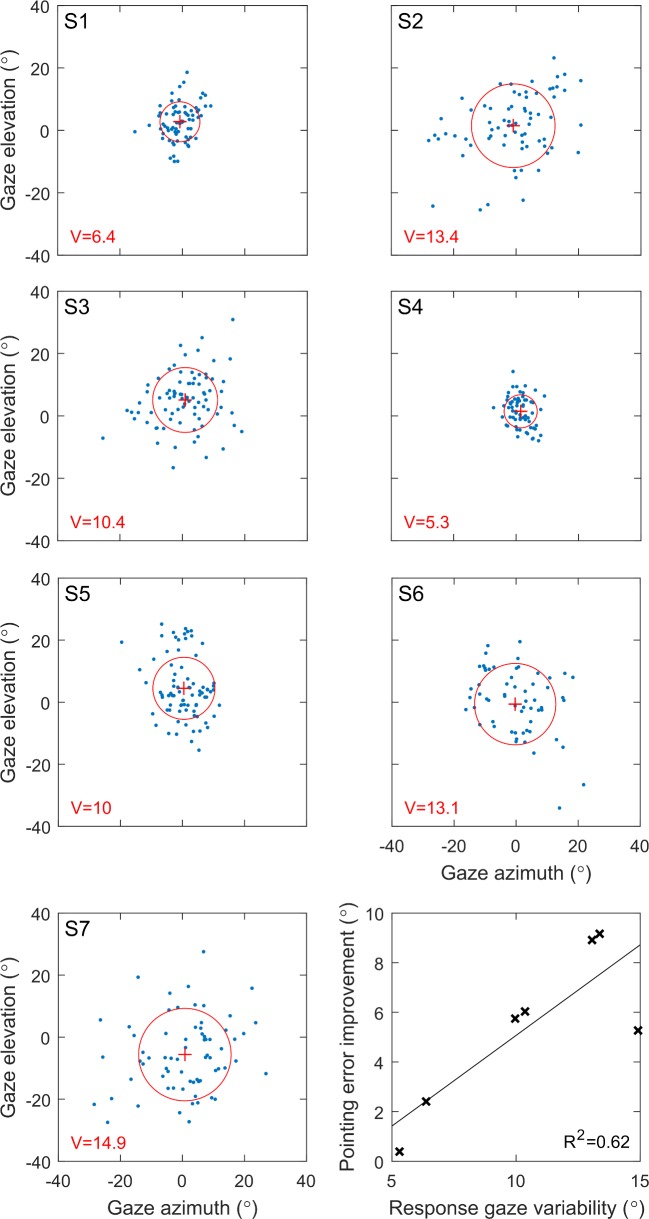
Greater variability in uncompensated response gaze is correlated with an improvement in pointing accuracy in the center-fixed condition, demonstrating that compensation for variable response gaze provides this benefit. Panels S1 to S7 show the gaze position at time of response for trials in the uncompensated condition for each subject, with the centroids denoted by red plus symbols. The variability of the distribution (red circle) is characterized by V, the mean distance from the centroid. Bottom right: difference in mean pointing error between the center-fixed and uncompensated conditions versus the response gaze variability in the uncompensated condition for each subject.

After completing the task, four subjects, S1, S2, S4, and S5, reported that they primarily used head scanning while attempting to keep their eyes fixed centrally. For S1, S2, and S4 this was because they had noticed that the percept was moving in response to their eye movements, while S5 could not articulate a reason. S3, S6, and S7 reported using head and eye scanning in conjunction. The distribution of response gazes in the uncompensated condition is plotted for each subject in Figure 8. Each point represents the gaze at the time of response for a single trial, and the variability of the distribution was characterized by the mean distance from the centroid, *V*. Greater variability was correlated with greater difference in average pointing error between the center-fixed and uncompensated conditions (*R*^2^ = 0.64).

### Range of Eye Movements Required During Gaze Compensation

A gamma function was fitted to the distribution of eye position magnitudes for all eye position recordings made during trials in the gaze-compensated condition. Data from all subjects was analyzed collectively. Based on the cumulative distribution function of the fitted distribution, 95% of the measured eye positions were within 24.44° of the natural gaze origin.

## Discussion

The present study tested the effectiveness of gaze compensation in improving hand-eye coordination in a target localization task under simulated prosthetic vision. Gaze compensation significantly reduced pointing error in six of seven subjects when compared with the uncompensated condition. Further, pointing error in the gaze-compensated condition was similar or better for all subjects when compared with that in the center-fixed condition in which phosphenes did not move. The gaze at the response time was found to be predictive of the direction of pointing error, and more eccentric gaze was predictive of higher magnitude pointing error. These results strongly suggest that noncentral gaze impeded coordination in the uncompensated condition by misrepresenting the location of the target and biasing pointing direction towards the gaze point.

Four of seven subjects reported that they primarily relied on head scanning and attempted to confine their gaze to the central region. This strategy is the same as the standard instruction given to Argus II recipients to minimize the incidence of camera-gaze misalignments. Interestingly, the subject with the least variability in response gaze (S4, Fig. 8) was the same subject for whom the pointing error did not depend on the condition tested. This can be attributed to a floor effect; eye movements were suppressed sufficiently well that any negative effect of gaze on coordination was beneath the noise floor. The remaining six subjects were less successful in suppressing eye movement and all benefited from gaze compensation. Further, greater variability in response gaze was correlated with greater reduction in pointing error in the gaze-compensated condition compared with the uncompensated condition. This result highlights the importance of suppressing eye movements in prosthetic vision; however, it is notable that the majority of subjects were unsuccessful in suppressing eye movement. This observation is in agreement with the existing literature, which reports that even experienced retinal implant recipients have difficulty suppressing eye movement.^8,34^ In contrast, gaze compensation provided coordination comparable to the center-fixed condition for all subjects with no training or suppression of eye movement required. The findings provide motivation for the integration of eye trackers into visual prosthesis devices for gaze compensation.

### Response Time

McIntosh^23^ reported that subjects were faster at a visual search task when gaze compensation was used, while Sabbah et al.^8^ reported that patients implanted with the Argus II often made a series of time-consuming compensatory head and neck movements to resolve camera-gaze misalignments before reaching for an object. In contrast, the present study showed significantly decreased response times in the gaze-compensated condition for only one of seven subjects. Two aspects of the experiment design may have contributed to this. First, subjects were not briefed on the simulator conditions or told which condition was active at any time. Second, there was no visual or tactile feedback with which the subject could gauge their pointing accuracy. Thus, the subjects had no opportunity to develop compensatory techniques for any specific condition.

### Gaze Correlated With Pointing Error

Previous studies have revealed that a relationship between eye movement and pointing error exists,^8,25^ but to our knowledge the present study is the first time that relationship has been quantified. Interestingly, the linear correlations that were observed between gaze and pointing error in the uncompensated condition had gradients less than one (Fig. 7). In other words, pointing error was on average less eccentric than gaze, even though the percept was displaced by an amount equal to the gaze angle. This is in agreement with Endo et al.,^33^ who reported that “low vision subjects tended to touch more toward the central side of the target.”

### Limitations of Simulated Prosthetic Vision

The prosthetic vision simulation paradigm used in this study was idealized compared with present visual prostheses. In particular, the simulated phosphenes were uniform in shape and layout, unlike the phosphenes elicited by stimulation of the retina, which are known to be irregular in size, shape, and layout.^32^ It is therefore likely that the resolution of the simulated prosthetic vision was higher than that of present devices. However, the optotypes used in the task were relatively large and simple, and subjects were not required to resolve any particular detail of the optotype, only to detect its presence and location. We should also consider that practiced implantees might possess a certain level of intuition in interpreting phosphenated vision that was not available to the healthy-sighted subjects in this study, and that additional confounding factors (such as irregular phosphenes) might have obscured the result while being only tangential to the hypothesis. The results are consistent with findings in actual prosthesis recipients (Caspi et al. *IOVS* 2017;58:ARVO E-Abstract 4192) and simulated prosthetic vision (Hozumi et al. *IOVS* 2016;57:ARVO E-Abstract 1958), and we therefore find it unlikely that the uniform phosphenes of the simulator imparted any significant advantage. The findings of this simulation study are also likely to extend to future high-resolution devices.

### Implementation of Gaze Compensation in Visual Prostheses

Selecting the correct region of the image to display to the patient requires accurate calibration of the eye tracker and consideration of the surgical placement of the electrode array, which may be eccentric from the fovea. Typical fixation target eye tracker calibration routines are inaccessible to visual prosthesis patients, who lack foveation, so unconventional calibration techniques are necessary. Caspi et al.^25^ demonstrated a calibration routine for retinal implants, whereby patients repeatedly placed a visual marker at arm's length to indicate the location of a percept. The authors solved for the calibration coefficients and the retinotopic placement of the electrodes by assuming a 1:1 correlation between gaze displacement and the eccentricity of the marked location.

The notion that pointing tasks can be a useful tool for eye tracker calibration is supported by the linear relationship between gaze and pointing error presented in this study; however, pointing error was on average smaller than gaze eccentricity (Fig. 7, uncompensated condition: gradients <1). It follows that a calibration dependent upon patients pointing to a percept may underestimate gaze angles. Other biases in pointing direction may also affect the calibration, such as the downward and lateral bias in open-loop pointing that exists in normal vision^33^ and in artificial vision.^8^ The role of pointing in calibration could be minimized by instead requiring patients to move their eyes so as to align the percept with a tactile target, though the accuracy of such eye movements and the ease with which they can be executed has, to our knowledge, not been addressed in the literature. Passive calibration procedures that determine the geometry of the eye without requiring cooperation from the patient may also be useful.^35^

A second consideration for the clinical implementation of gaze compensation is that the range of measurement of the eye tracker should sufficiently encompass the normal range of movement of the eye. Gaze was found to be within 24.44° of the natural gaze origin 95% of the time during gaze-compensated viewing. Therefore, a range of measurement of ±25° would be a reasonable minimum specification for an eye tracker for retinal implants. This is within the specifications of modern video-based eye trackers.^36^ Kanda et al. have demonstrated tracking the rotation of the eye by measuring stimulus artifact through electrodes implanted in the canthus, but did not report the range of measurement of their system (Kanda et al. *IOVS* 2017;58:ARVO E-Abstract 4188). It should be noted that eye movements are known to be partly driven by visual stimulus,^37,38^ and patients with retinitis pigmentosa are known to exhibit different oculomotor behavior in certain tasks when compared with healthy-sighted subjects.^39–41^ Therefore, in practice the range of eye movement of a visual prosthesis recipient may differ from that observed here. It may also be possible that horizontal range of measurement is more valuable than vertical range of measurement in realistic scenarios in which horizontally arranged visual information and horizontally moving objects are prevalent. Other considerations that may be important are the increased weight, power consumption, and processing latency that eye tracking apparatus would add to a visual prosthesis.

### Conclusion

In conclusion, gaze compensation was effective in improving hand–eye coordination in a target localization task. Eccentric gaze was found to be correlated with poor coordination under prosthetic vision simulation that modeled retinally stabilized phosphenes. The results emphasize the potential for eye trackers to improve patient outcomes in prosthetic vision.
